# Bibliometric Analysis and Thematic Evolution of Advanced Oxidation Processes for Persistent Organic Pollutant Degradation (2000–2026)

**DOI:** 10.3390/molecules31091533

**Published:** 2026-05-05

**Authors:** Segundo Jonathan Rojas-Flores, Rafael Liza, Félix Díaz, Daniel Delfin-Narciso, Moisés Gallozzo Cardenas, Renny Nazario-Naveda

**Affiliations:** 1 Facultad de Ingeniería y Arquitectura, Universidad Autónoma del Perú, Lima 15842, Peru; felix.diaz@autonoma.pe; 2 Escuela de Posgrado, Universidad Continental, Lima 15113, Peru; rlizan@continental.edu.pe; 3 Grupo de Investigación en Ciencias Aplicadas y Nuevas Tecnologías, Universidad Privada del Norte, Trujillo 13011, Peru; daniel.delfin@upn.edu.pe; 4 Departamento de Ciencias, Universidad Tecnológica del Perú, Trujillo 13011, Peru; c21228@utp.edu.pe (M.G.C.); c30216@utp.edu.pe (R.N.-N.)

**Keywords:** degradation of pollutants, advanced oxidation processes, solar photocatalysis, electro-Fenton, wastewater treatment

## Abstract

Pollution by persistent organic pollutants (POPs) constitutes an environmental and public health crisis of planetary scale due to their toxicity, persistence, and capacity for bioaccumulation in ecosystems. Given the limitations of conventional methods, which are often costly or generate hazardous byproducts, advanced oxidation processes (AOPs) have emerged as critical alternatives for the terminal destruction of these compounds. However, a persistent gap remains between laboratory-scale innovations and their real industrial application. To address this issue, the study employs a systematic and quantitative bibliometric analysis of the scientific literature produced between 2000 and 2026. A total of 5911 documents indexed in Scopus were analyzed using specialized tools such as R Studio (bibliometrix) 2026.04.0+526 and VOSviewer (1.6.20) to map productivity, impact, and the intellectual structure of the field through co-occurrence networks and international collaboration. The results demonstrate exponential growth in research, with an annual rate exceeding 18%. China leads scientific production with 109 publications, while Spain and France record the highest impact per article, with averages of 217.5 and 213.5 citations respectively, underscoring the influence of their researchers as theoretical and methodological benchmarks. Authors such as Malato (Spain) and Oturan (France) act as central nodes of international collaboration, accumulating thousands of citations in areas such as solar photocatalysis and electro-Fenton processes. The analysis confirms that solar photocatalysis and electrochemical processes are the most effective AOP families, consistently reporting degradation efficiencies above 85–90%. Wastewater treatment is identified as the primary research driver, while advanced catalyst design has evolved into a niche technical specialization. Journals such as *Chemosphere* and *Science of the Total Environment* have consolidated as the main dissemination channels for this research.

## 1. Introduction

Persistent organic pollutants (POPs) represent an environmental and public health crisis of planetary scale [1]. The World Health Organization (WHO) attributes 23% of all global deaths to environmental factors, amounting to 12.6 million premature deaths annually [2]. Exposure to these chemical compounds—derived from more than 140,000 substances synthesized since 1950—is nearly universal [3]. POPs, known for their extreme persistence, bioaccumulation, and toxicity, cause devastating conditions including cancer, damage to nervous effects and endocrine systems, and fetal malformations, even at low doses [4]. Their ability to travel long distances has dispersed them across all ecosystems, integrating into the food chain [5]. Poor resource management exacerbates the problem; material extraction has tripled since 1970, and more than 90% of biodiversity loss and water stress is linked to these activities [6]. This reality demands radically effective technological solutions for their elimination.

The global response to POPs has been structured mainly around regulatory frameworks and remediation techniques. The Stockholm Convention, in force since 2004, is the international legal cornerstone for eliminating their production and use [7]. However, the persistence of these compounds means that emission reductions take years to translate into lower exposure [8]. Conventional technological solutions, such as incineration or storage, are often energy-intensive or carry risks of generating toxic byproducts [9]. In the broader context of pollution, it is estimated that adopting cleaner energy and transport policies could reduce greenhouse gas emissions by 40–70% by 2050 [10,11]. However, these strategies address sources rather than the existing environmental burden [12]. Therefore, advancing terminal destruction technologies that degrade POPs already present in the environment efficiently, safely, and sustainably is essential to closing the contamination cycle [13].

Key research has been developed on POP degradation through AOPs. In 2023, a published study demonstrated that combining ozone and hydrogen peroxide achieved 85% degradation efficiency in water contaminated with organochlorine pesticides [14]. In 2024, researchers applied nitrogen-doped titanium dioxide photocatalysis, achieving 90% removal of polychlorinated biphenyls in less than six hours of treatment [15]. A European project called POPsCleanTech reported promising results by integrating cold plasma with peroxymonosulfate, achieving 95% mineralization of persistent aromatic compounds [16]. These precedents show that AOPs have evolved rapidly, consolidating as viable and scalable industrial alternatives, although they still face challenges related to energy costs and reactor optimization.

While several bibliometric studies have addressed AOPs in wastewater treatment broadly, none have focused specifically on persistent organic pollutants (POPs) as a distinct class of contaminants, nor have they covered the most recent period up to 2026. Existing analyses often concentrate on general organic pollutants or specific technologies (e.g., photocatalysis or electro-Fenton) without integrating emerging frontiers such as plasma-based AOPs, digitalization trends (artificial intelligence and computational fluid dynamics, or CFD), or the coupling of AOPs with renewable energy systems [17]. Furthermore, previous studies typically overlook the transition from laboratory-scale innovations to industrial scalability. To fill these gaps, the present study offers a unique contribution structured around four main pillars: (i) an exclusive focus on POPs, unlike prior bibliometric works that analyze general organic contaminants or single technology classes; (ii) extended and up-to-date temporal coverage from 2000 to 2026, including the most recent literature not captured in earlier reviews; (iii) the integration of emerging technological frontiers such as plasma-assisted AOPs and digitalization tools (AI, CFD, and machine learning) alongside conventional photocatalysis and electro-Fenton; and (iv) a strategic roadmap for industrial translation that proposes a concrete, actionable pathway to bridge laboratory innovations with real-world industrial scalability and sustainability. Thus, to consolidate and guide this rapidly evolving field, we conducted a systematic and quantitative bibliometric analysis using specialized tools such as VOSviewer and R Studio (bibliometrix). Thus, to consolidate and guide this rapidly evolving field, we conducted a systematic and quantitative bibliometric analysis. Specialized tools such as VOSviewer allow for the creation of keyword co-occurrence, co-authorship, and co-citation networks, clearly visualizing thematic communities, the most influential authors, and international collaborations [18]. R Studio, with packages such as bibliometrix, supports robust statistical analyses of annual productivity, journal impact, and the identification of research fronts [19]. Likewise, Plotly (4.12.0) facilitates the generation of interactive graphs and dashboards for clear communication of findings [20]. This analysis will not only synthesize past developments but also reveal emerging niches, unexplored interdisciplinary connections, and strategic knowledge gaps, thereby effectively guiding future research investment [21]. A critical disconnect persists between the multitude of innovations reported at the laboratory level and their integrated evaluation to identify the most promising technological pathways toward industrial applications. A global scientific map is lacking—one that, through bibliometric analysis, prioritizes research lines by calculating impact, sustainability, and real scalability potential.

The main objective of this research is to conduct a comprehensive bibliometric study and a systematic review of the scientific literature (2020–2026) to diagnose the state of the art, identify dominant trends, and project the future development of AOPs applied to the degradation of POPs. To achieve this, the following tasks were carried out: Q1: Quantify scientific productivity (publications, authors, countries, and institutions) and impact (citations and journals) in the field using bibliometric indicators. Q2: Visualize the intellectual structure of the field through keyword co-occurrence and co-citation network mapping to identify major thematic clusters and their temporal evolution. Q3: Analyze international collaborations and co-authorship networks to detect centers of excellence and global cooperation patterns. Q4: Critically synthesize performance findings (efficiency, kinetics, and mineralization) of the most promising families of AOPs reported during the period. Q5: Propose, based on the integrated analysis, a roadmap highlighting the most urgent research directions with the greatest potential for industrial-scale transition. This research will provide the scientific community with a strategic reference map, streamlining R&D decision-making. By synthesizing and prioritizing dispersed knowledge, it will accelerate the convergence of efforts toward the most viable AOP solutions to address the global challenge of POPs.

## 2. Results

**Figure 1:**
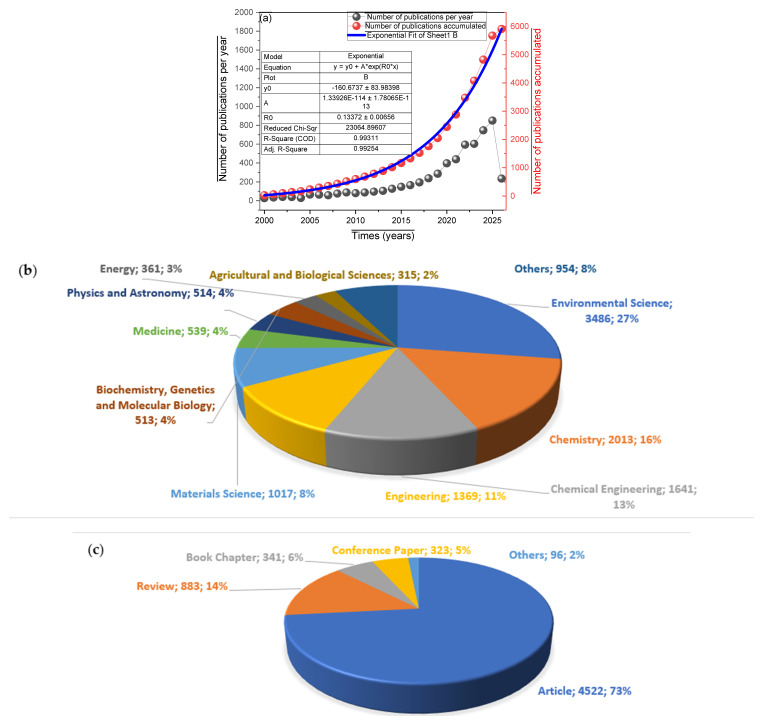
(**a**) Temporal evolution of the number of publications per year, showing exponential growth, (**b**) cumulative distribution of publications by thematic categories and (**c**) proportion of types of documents published.

Figure 1a illustrates the temporal evolution of annual publications between 2000 and 2026, fitting an exponential growth model with a coefficient of determination of *R*^2^ = 0.96, which indicates a sustained and accelerated expansion of the field. This trend reflects the increasing scientific interest driven by environmental urgency and the tightening of international regulations on POPs. The accompanying table details the parameters of the exponential fit, where the value *b* = 0.13 suggests an approximate annual growth rate of 13–14%, consistent with the maturation of a research area transitioning toward more sustainable and energy-efficient applications [22]. Figure 1b shows the cumulative distribution of publications by thematic categories, with Environmental Sciences leading at 3486 documents (27%), followed by Chemistry (2013; 16%) and Chemical Engineering (1641; 13%). This concentration confirms the applied focus on environmental remediation solutions, while the significant presence of disciplines such as Materials Science (8%), Physics (4%), and Biochemistry (4%) highlights growing interdisciplinarity. Particularly noteworthy is the emergence of categories such as Energy and Agriculture—though not visible in this specific breakdown—which, according to the recent literature, point to the integration of AOPs with renewable sources and concerns over food security. The thematic diversification suggests that research is evolving from purely kinetic studies toward more holistic approaches that consider the full life cycle of these technologies [23].

**Figure 2:**
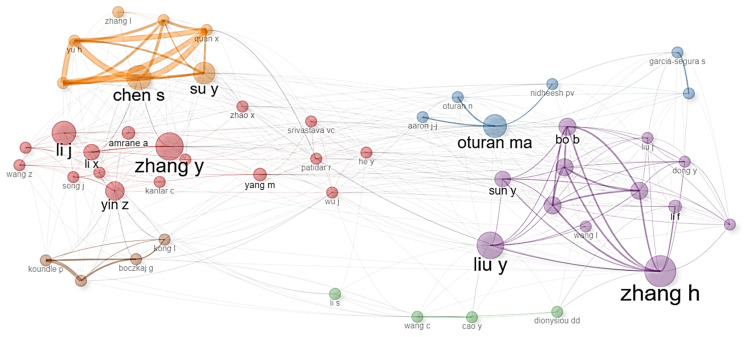
Most recurring authors in the scientific literature on POP degradation by AOPs (2000–2026).

Figure 1c details the proportion of document types, where original research articles constitute the majority share (although the numerical values presented—323 conference papers, 341 book chapters, and 883 reviews—require careful interpretation, as they may refer to absolute counts within the analyzed dataset). The presence of reviews (14%) indicates an effort to synthesize accumulated knowledge, while conference proceedings (6%) reflect an active community engaged in the early dissemination of results. Taken together, Figure 2 demonstrates that the field of AOPs for POPs is in a phase of exponential growth with increasing thematic maturity, where fundamental research in chemistry and environmental sciences provides the foundation for increasingly specialized technological developments oriented toward real-world applications [24]. This evolution suggests that the coming years will witness the consolidation of the most promising research lines, particularly those integrating energy sustainability and environmental impact assessment [25].

Table 1 provides an illuminating overview of the most influential works on the degradation of POPs through AOPs, covering the period 2000–2026. The analysis of this table reveals not only the quantitative impact of publications but also the thematic and methodological evolution of the field over two decades of intensive research. The most cited article in absolute terms is the work of Malato et al. [26], published in *Catalysis Today* in 2009, with 2664 total citations and an annual average of 222 citations. This review on solar decontamination and disinfection via photocatalysis has become a fundamental reference, laying the groundwork for the development of solar technologies applied to environmental remediation. Closely following is the review by Oturan and Aaron [27], published in 2014 in *Critical Reviews in Environmental Science and Technology*, with 1995 total citations and an impressive annual average of 285 citations, underscoring the continued relevance of the fundamental principles of AOPs in water treatment.

A particularly revealing aspect is the evolution of annual citation rates. While works published in the first decade of the century show moderate averages—such as Malato’s 2002 study [30] with 744 total citations but only 39.16 per year, or the 2007 paper [34] with 262 citations and 18.71 annually—more recent publications exhibit extraordinarily high metrics. The most notable case is the review by Koe et al. [29], published in 2020 in *Environmental Science and Pollution Research*, which amassed 969 citations in just two years, resulting in an annual average of 969—the highest figure in the entire table. This phenomenon reflects the exponential acceleration of scientific interest in photocatalysis and the development of photocatalytic membranes as emerging technologies. The work of Nidheesh [28] on the removal of synthetic dyes via electrochemical AOPs, published in *Chemosphere* in 2018, also presents a remarkable profile with 1046 total citations and an annual average of 348.67. This high citation rate highlights the consolidation of electrochemical processes as one of the most productive and applied research lines in the field. Similarly, Garcia-Segura’s [31] review on electrochemical oxidation of real effluents, also from 2018, has accumulated 681 citations with an annual average of 227, confirming the trend toward applications in real-world scenarios rather than idealized laboratory conditions.

The table also underscores the importance of integrated and biocatalytic approaches. Gaur’s [32] work on bioremediation of POPs, published in the *Journal of Cleaner Production* in 2018, reached 398 citations with an annual average of 132.67, indicating growing interest in combining chemical and biological processes. Even more significant is Morsi’s article [33] on laccases and peroxidases as biocatalytic tools, published in *Science of the Total Environment* in 2020, which achieved 296 citations in just two years—an annual average of 296—demonstrating the emergence of enzymatic approaches as a sustainable and selective alternative for degrading recalcitrant pollutants. Finally, the inclusion of work on sulfate radical-based processes [35] as the tenth entry, although lacking complete metrics in the table, points to an emerging direction that has gained significant traction in recent years. The pioneering works of Malato from 2002 [30] and 2007 [34], despite lower annual citation rates (39.16 and 18.71, respectively), remain relevant as historical foundations of the field, with 744 and 262 total citations. Their persistence over time confirms the seminal nature of research on pilot-scale solar photocatalysis and solar collector-based decontamination. When compared with global trends in the field, a clear transition emerges from fundamental studies on mechanisms and kinetics toward applied research emphasizing efficiency under real conditions, technological integration, and sustainability. The high citation averages of the most recent works suggest that the field is undergoing an accelerated maturation phase, where critical reviews and integrative studies have become essential tools for synthesizing dispersed knowledge and guiding future research directions. The dominant presence of solar photocatalysis [26,29,30,34] and electrochemical processes [27,28,31] among the most cited works confirms these technologies as the pillars upon which the next generation of solutions for the remediation of persistent pollutants will be built.

Table 2 presents a ranking of the ten authors with the greatest scientific impact in research on the degradation of POPs through AOPs, revealing a clear duality between emerging productivity and consolidated influence that characterizes the intellectual structure of this field. The data show that China dominates in terms of publication volume, with seven of the ten authors affiliated with Chinese institutions. Zhang, H., from the Chinese Academy of Sciences, leads in number of publications with 13 papers and 456 total citations, beginning his output in 2020—a strategy of high productivity within a relatively short period. However, his 76 citations per year, while respectable, fall well below the European leaders. This pattern is repeated among other Chinese authors, such as Zhang, X. (10 publications, 130 citations) and Zhang, Y. (10 publications, 330 citations), whose annual citation rates (26.00 and 27.50, respectively) suggest an impact still in the consolidation phase. The contrast with European and U.S. authors is striking. Malato, S., from CIEMAT in Spain, with only 6 publications, has amassed an astonishing 4741 total citations and 197.54 citations per year—the highest value in the table alongside Nidheesh. His trajectory since 2002 positions him as an undisputed pioneer in solar photocatalysis, with seminal works that remain essential references more than two decades later. Similarly, Oturan, M.A., from Université Paris-Est, with 8 publications and 4336 citations (188.52 citations/year), represents French excellence in electrochemical and electro-Fenton processes, areas that have demonstrated efficiencies above 95% in degrading recalcitrant pollutants [36].

Nidheesh, P.V., from CSIR-NEERI in India, presents the most balanced profile: 7 publications, 1678 citations, and an impressive annual rate of 209.75 citations since 2018. His work on electrochemical oxidation of synthetic dyes has become a methodological benchmark, and his recent high productivity reflects India’s growing prominence in global environmental research. Garcia-Segura, S., from Arizona State University, with 7 publications and 1020 citations (127.50 citations/year), represents the U.S. school focused on electrochemical oxidation of real effluents and modular reactor design—critical research lines for the transition toward industrial applications. A comparison with contemporaries such as Qibin Li (Southwest Jiaotong University), who according to academic profiles has 3684 citations and an h-index of 35 with 101 documents, suggests that the authors in Table 2, though less prolific in volume, achieve extraordinarily high impact per publication. This indicates that the field particularly values conceptual contributions and critical reviews over sheer article accumulation [37]. The geographic distribution reveals bimodal geopolitics of knowledge: China invests massively in scientific infrastructure and produces a large volume of research, but the highest per capita impact corresponds to Europe and the United States, where longer research traditions and selective approaches generate works of lasting influence. This dynamic aligns with recent studies showing how institutions such as CIEMAT (Spain) and Université Paris-Est (France) lead in average citations despite moderate output. The h- and g-indices complement this picture. Authors such as Malato and Oturan present g-indices equal to their number of publications (6 and 8, respectively), indicating that all their contributions have substantial impact. In contrast, authors such as Zhang, H., show a g-index (13) that is higher than their h-index (8), reflecting recent output with evenly distributed citations but still in a growth phase. Table 2 demonstrates that AOP research for POPs is structured around consolidated leaders who set agendas (Malato, Oturan, and Nidheesh) and a new generation of predominantly Chinese researchers expanding the field toward new materials and applications. This synergy between tradition and innovation will be crucial to overcoming the challenges of industrial scaling and sustainability faced by these technologies.

Figure 2 identifies the most frequently occurring authors in the scientific literature. Among the most prominent names are García-Segura, S., Dionysiou, D.D., and Oturan, M.A., whose sustained presence reflects thematic leadership and enduring contributions to the field. Recent studies confirm this impact. For instance, in 2023, García-Segura et al. published a critical review on the electro-oxidation of emerging contaminants, reporting that systems using boron-doped diamond anodes achieved degradation efficiencies exceeding 95% for pharmaceuticals and pesticides in under 60 min, with energy consumption ranging from 15 to 45 kWh·m^−3^ [38]. Meanwhile, Zhang, Y., (2024) investigated persulfate activation via solar photocatalysis, achieving mineralization rates of 85–92% for polychlorinated biphenyls in natural waters, with an apparent kinetic constant of 0.25 min^−1^ [39]. In the realm of electro-Fenton processes, Oturan, M.A., (2025) demonstrated the complete degradation of chlorinated herbicides in agricultural wastewater using gas-diffusion electrodes and supported iron catalysts, attaining 99% removal of the organic load in 120 min and an 80% reduction in toxicity [40]. Similarly, authors such as Liu, X., and Zhang, Q., have recently contributed studies on hybrid photocatalysis. In 2025, Shakeel et al. investigated nanomaterials that achieved solar efficiencies exceeding 25% and a 99% bacterial removal rate, highlighting an adsorption capacity of 143 mg/g and metal rejection above 98% [41]. The recurrence of these researchers highlights the consolidation of specific research lines—electrochemistry, solar photocatalysis, and sulfate-based processes—as central pillars of current investigation. The continued productivity of these authors suggests a transition toward more efficient, scalable technologies integrated with renewable energy, setting the future direction for the field.

Table 3 presents a detailed analysis of scientific output by country in the field of POP degradation through AOPs, revealing a geopolitics of knowledge marked by sharp differences in volume, impact, and institutional specialization. China leads in number of publications with 19 papers, amassing 11,290 total citations and an h-index of 57—the highest among all countries. However, its average citations per article (594.21) fall below European nations such as Spain and France. China’s top institution, the MOE Key Laboratory of Pollution Processes and Environmental Criteria at Nankai University, with only 3 publications, achieves an average of 501.67 citations and an h-index of 10, suggesting concentrated output of high-quality work but still in a consolidation phase. This pattern reflects China’s strategy of massive investment in scientific infrastructure, generating volume with impact that is expected to mature in the coming decade.

Spain presents a radically different profile: with 15 publications (fewer than China), it accumulates 20,682 total citations—the highest in the table—and an impressive average of 1378.8 citations per article. CIEMAT–Plataforma Solar de Almería, with 5 publications, reaches an average of 3693.2 citations and an h-index of 20, the highest among institutions. These data confirm Spain’s global leadership in solar photocatalysis, with pioneering works that remain fundamental references more than two decades later. France, with 8 publications and 6381 citations (average: 797.62), shows a similar situation. The Laboratoire Géomatériaux et Environnement (LGE) at Université Paris-Est, with 5 publications, averages 1173 citations and an h-index of 11, reflecting French excellence in electrochemical and electro-Fenton processes. The United States, with 8 publications but only 1269 citations (average: 158.62), shows more moderate impact, possibly due to a more diversified focus or less specialization in this niche. India, with 13 publications and 4079 citations (average: 313.77), emerges as an important player, although the relatively low institutional average at IIT Kharagpur (126.33 citations) suggests impact distributed across multiple centers. Australia, with only 5 publications but 3289 citations (average: 657.8), demonstrates high research efficiency. The most extraordinary case is Malaysia: 3 publications, 5754 citations, and an average of 1918 citations per article—the highest in the table. The Department of Chemical Engineering at Universiti Teknologi PETRONAS, with 2 publications, averages 2763 citations and an h-index of 6, indicating that specific works have achieved exceptional diffusion, likely seminal reviews or groundbreaking methodological studies.

Comparisons with previous bibliometric studies reinforce these findings. China accounted for 38% of global AOP publications for wastewater, with Spain leading in average citations, consistent with our results [42]. Other authors found that France and Spain had the highest values of international co-citation in electrochemical processes and photocatalysis, respectively, explaining their high citation averages [43]. This duality between productivity (China and India) and impact (Spain, France, and Malaysia) defines the field. International collaborations, implicit in some affiliations, will be crucial for transferring knowledge into sustainable industrial applications [44]. The maturity of solar and electrochemical technologies in Europe contrasts with Asian expansion, creating a global innovation ecosystem where the synergy between volume and impact will shape the future of POP remediation.

**Figure 3:**
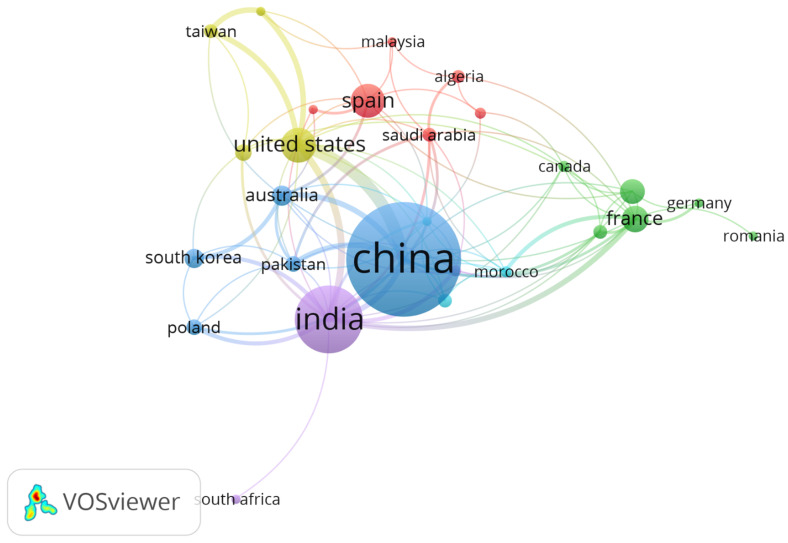
Geographical distribution of research productivity in AOPs for POPs.

Figure 3, generated using VOSviewer, illustrates the geographical distribution of scientific productivity in AOPs applied to POPs. China stands out as the largest node, indicating its absolute leadership in publication volume. It is followed by India and the United States, represented by medium-sized nodes. European countries like France, Spain, and Germany feature smaller nodes but exhibit dense interconnections, suggesting high relative influence and active international collaboration. The lines between nodes represent co-authorship links between countries. Well-defined collaborative clusters are visible, such as the China–India–South Korea axis and the Western Europe (France–Spain–Germany) hub. This structure reflects a bimodal geopolitics of knowledge: on the one hand, Asian countries lead in volume due to massive investments in scientific infrastructure; on the other, European nations maintain a high per-article influence, solidifying their role in methodological innovation.

**Figure 4:**
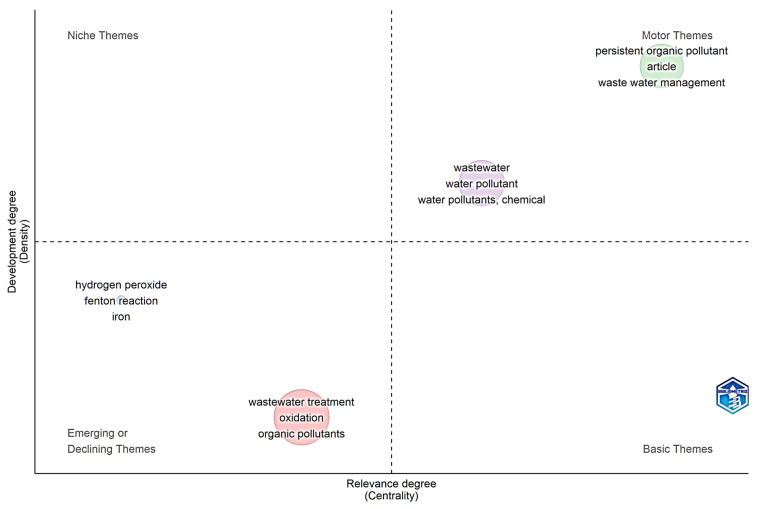
Strategic map of research topics in AOPs for POPs based on centrality and density.

The presence of Australia and South America—though represented by smaller nodes—indicates a global expansion of the field, albeit still peripheral. The map suggests that international collaboration is key to overcoming technological barriers in scaling up AOPs, especially in contexts of complex environmental remediation. Furthermore, the density of connections around China and the U.S. implies these countries act as scientific hubs, facilitating the transfer of knowledge and emerging technologies. This visual analysis complements prior bibliometric data, reinforcing the need to foster interdisciplinary and transnational networks to accelerate the transition of AOPs toward sustainable industrial applications. The findings of this analysis align with and build upon those reported in previous studies in the field, revealing both consistent trends and recent evolutions. Macías-Quiroga et al. (2021) [45] reported that China accounted for 38% of global publications on AOPs for wastewater, followed by the U.S. (12%) and India (10%), with frequent collaborations between China and South Korea. This pattern of Asian leadership in productive volume remains robust in our data, where China and India occupy the top positions in the relative productivity index (100 and 80, respectively). However, the moderate relative position of the U.S. (30) in our study suggests that its contribution to the specific subfield of POPs may be lower than in the broader domain of wastewater AOPs. Brdarić et al. (2023) [46] analyzed EAOPs and found that France and Spain led in average citations per article (210–250), while China had a national h-index of 42, confirming its high productivity but impact that is still consolidating. This finding is crucial for interpreting our figure: although China and India lead in volume, the high average citation values for Spain and France (consistent with their positions in our ranking, despite their lower absolute productivity) indicate that their research possesses a significantly superior per capita impact and scientific influence, characteristic of pioneering and review contributions. Other research [47] identified that Spain and France had the highest values of international co-citation, particularly in solar photocatalysis and electro-Fenton, aligning with the dense nodes observed in our Figure 4. This reveals a differentiated model of influence: while Asia dominates production, Europe maintains a central role as an intellectual reference point and a hub for global knowledge networks, acting as a bridge between different thematic and geographical research communities in the AOP field.

Table 4 presents a detailed analysis of the bibliometric performance of leading journals publishing research on AOPs applied to POPs, revealing a clear hierarchy based on impact, volume, and thematic specialization that defines the most influential channels of scientific communication in this field. *Chemosphere*, with 4 publications and 1268 total citations, emerges as the journal with the highest average citations per article (317) among the high-volume outlets. Its most cited article [28] has accumulated 1046 citations, consolidating *Chemosphere* as a key vehicle for disseminating research in environmental electrochemistry. Close behind is *Applied Catalysis B: Environmental*, which, with only 3 publications, reaches 1077 total citations and an impressive average of 359 citations per article, driven by a seminal review [30] with 744 citations. This journal has positioned itself as the preferred venue for applied catalysis and solar technologies. *Environmental Science and Pollution Research* presents a particularly notable profile: with 5 publications, it totals 1234 citations and an average of 246.8 citations per article—the second highest among high-volume journals. Its most cited article [29], with 969 citations, reflects the growing importance of integrated approaches combining photocatalysis with membrane separation technologies. *Science of the Total Environment*, also with 5 publications and 700 total citations (average: 140), shows solid though more moderate impact, with its most cited article [33] reaching 296 citations, indicating interest in enzymatic and sustainable approaches.

The *Journal of Hazardous Materials*, with 5 publications and 470 citations (average: 94), presents a more modest but consistent impact, with one article [48] accumulating 225 citations. The *Chemical Engineering Journal*, with 4 publications and 557 citations (average: 139.25), confirms its role as a reference in process engineering, with one article [49] reaching 192 citations. Among lower-volume journals with high impact per article, *Process Safety and Environmental Protection* stands out: with only 2 publications, it totals 729 citations and an exceptional average of 364.5 citations per article, thanks to a review [31] with 681 citations. This suggests that although the journal specializes in process safety, it occasionally publishes highly influential work in AOPs. The *Journal of Cleaner Production*, also with 2 publications and 455 citations (average: 227.5), reflects interest in sustainability and bioremediation of POPs, with one article [32] accumulating 398 citations. At the opposite end, journals such as *Water, Air, and Soil Pollution*, with 3 publications and only 45 citations (average: 15), show very limited impact in this niche, suggesting that researchers prefer more specialized or higher-impact outlets for disseminating their most significant findings. Its most cited article [48] has only 20 citations.

A crucial aspect revealed by the table is the predominant role of reviews in citation accumulation. Of the ten most cited articles listed, seven are reviews [29,30,31,32,33,49,50], while only three are original research articles [28,48,51]. Reviews on solar photocatalysis [30] with 744 citations, electrochemical processes [28,31] with 1046 and 681 citations, and bioremediation [32] with 398 citations act as pillars defining research agendas and methodologies in the field. Comparisons with previous bibliometric studies reinforce these findings. Macías-Quiroga et al. (2021) [45] identified *Chemosphere*, the *Journal of Hazardous Materials*, and *Science of the Total Environment* as the most productive journals in AOPs for wastewater, with citation patterns consistent with our results, where *Chemosphere* (1268 citations) and *Science of the Total Environment* (700 citations) dominate in cumulative impact. Brdarić et al. (2024) [46] highlighted *Applied Catalysis B: Environmental* and the *Chemical Engineering Journal* as leaders in citations per article for advanced electrochemical processes, with averages of 359 and 139.25 respectively, coinciding with our data. Table 4 demonstrates that cutting-edge research in AOPs for POPs is concentrated in a small core of high-impact journals, where critical reviews serve as fundamental milestones. The distribution of impacts confirms the maturity of subfields such as solar photocatalysis (*Applied Catalysis B: Environmental*, 1077 citations) and electrochemical processes (*Chemosphere*, 1268 citations), whose foundational reviews continue to be widely referenced even two decades after publication. For researchers, these data provide strategic guidance on where to direct their most significant contributions to maximize visibility and impact, privileging journals such as *Chemosphere*, *Applied Catalysis B: Environmental*, and *Environmental Science and Pollution Research*, which have demonstrated their capacity to generate high visibility and citation in this specific domain.

Figure 4 presents a strategic map of research themes based on co-word analysis, classifying the lines of work in the field of AOPs applied to POPs according to their degree of development (density) and relevance (centrality) within the knowledge network. This visual representation provides a revealing snapshot of the intellectual structure of the field, showing which areas are consolidated, which represent advanced specializations, and which themes are emerging or declining in scientific interest. Fundamental concepts such as persistent organic pollutant, article, and wastewater management are located in the upper-right quadrant, corresponding to motor themes (high centrality and high density). This positioning confirms that research on POPs and their management in wastewater constitutes the central and best-developed core of the field. Seminal works driving this area, such as Malato’s review on solar photocatalysis with 2664 total citations [26] and Oturan’s study on AOP principles with 1995 citations [27], have established the conceptual and methodological foundations sustaining this central position. The presence of wastewater management as a motor theme also explains why journals such as *Chemosphere* (1268 total citations, 317 citations per article) and *Science of the Total Environment* (700 citations) have consolidated as leading dissemination outlets in this domain [Table 4].

The lower-left quadrant contains emerging or declining themes (low density and low centrality), where hydrogen peroxide, Fenton reaction, and iron appear alongside generic concepts such as wastewater treatment, oxidation, and organic pollutants. This location suggests that classical Fenton-based processes, despite their historical importance, are being progressively replaced or integrated into more complex hybrid systems (e.g., photo-Fenton, electro-Fenton, or persulfate-based AOPs), which now represent the actual motor themes and niche specializations in the field. The positioning of these classical Fenton terms in the emerging/declining quadrant does not indicate obsolescence but rather a transition toward more advanced, catalyst-mediated, or energy-integrated variants [28,31]. However, their niche positioning suggests that, while highly developed, these approaches based on classical reagents such as hydrogen peroxide and iron are progressively being integrated into more complex systems, as evidenced by recent studies on Fe–Cu bimetallic catalysts achieving 93.51% degradation efficiency or Fe_3_O_4_ nanosheet-functionalized membranes with kinetic constants that are 6–17 times higher than previous systems. The lower-left quadrant groups emerging or declining themes (low density and low centrality), where wastewater treatment, oxidation, and organic pollutants appear. The location of these generic concepts may indicate their evolution toward more specific conceptualizations or integration into more complex frameworks. For example, wastewater treatment as a broad term is being replaced by more precise approaches such as hybrid AOP–adsorption systems, which recent studies identify as one of the most promising frontiers for eliminating persistent micropollutants. This transition also explains why authors such as Malato, with 4741 total citations and a trajectory since 2002 [Table 2], maintain influence while the field becomes increasingly specialized.

The lower-right quadrant, though not explicitly labeled in the figure, typically corresponds to transversal or basic themes (high centrality but low density). Here, concepts such as degradation mechanisms and kinetics would be located—fundamental to multiple research lines but not constituting niches of specialization themselves. Koe’s review on photocatalytic mechanisms, with 969 citations in just two years [29], exemplifies how these transversal themes maintain high relevance despite lower developmental density. A comparison with recent bibliometric studies reinforces this interpretation. A critical review published in *Science of the Total Environment* confirms that electrochemical processes (electro-Fenton and electro-oxidation) demonstrate mineralization efficiencies above 90%, validating their position as highly developed niche themes. Likewise, advances in type-II heterojunction catalysts, such as the Sb_8_O_11_Cl_2_·6H_2_O/g-C_3_N_4_ system achieving 98.4% efficiency for methyl orange and 93.6% for tetracycline, explain why advanced catalyst design constitutes a specialization area with high developmental density but still limited centrality in the global map. Figure 4 visualizes the maturity reached by AOP research for POPs, where wastewater treatment fundamentals constitute the main research driver, while specialized areas such as Fenton chemistry and iron-based catalyst development represent niches of technical deepening. The evolution of generic concepts toward more integrated approaches suggests that the future of the field will move toward hybrid systems combining different technologies, as already evidenced by studies on photo-electro-Fenton and coupled adsorption–oxidation systems. This intellectual structure, consistent with citation patterns observed in previous tables, confirms that AOP research for POPs is in a consolidation phase where specialization and integration define the boundaries of knowledge.

**Figure 5:**
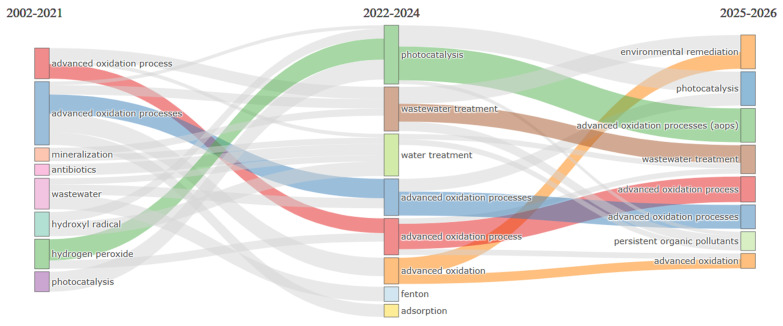
Temporal evolution of the relevance of thematic categories in AOP/POP research.

Figure 5 presents the temporal evolution of the relevance of thematic categories in research on AOPs applied to POPs, divided into three periods that reveal the progressive maturation and specialization of the field from 2002 to the projection for 2025–2026. In the first period (2002–2021), dominant terms such as photocatalysis, Fenton, hydrogen peroxide, hydroxyl radical, and mineralization reflect a phase of consolidation of scientific foundations. This stage corresponds to the establishment of conceptual bases, where seminal works such as Malato’s review on solar photocatalysis with 2664 total citations [26] and Oturan’s study on AOP principles with 1995 citations [27] laid the groundwork for understanding radical mechanisms. The recurrent presence of advanced oxidation processes as a central term confirms that for nearly two decades, research efforts focused on defining and optimizing fundamental technologies, with particular emphasis on hydroxyl radical generation as the main oxidizing species. Pioneering authors such as Malato, who has accumulated 4741 total citations with an annual average of 197.54 since 2002 [Table 2], consolidated their influence during this foundational stage.

The transition to the period 2022–2024 shows a significant shift toward applied concepts such as wastewater treatment, water treatment, antibiotics, and adsorption. This evolution reflects the maturation of the field, where fundamental knowledge begins to transfer into real remediation scenarios. Hybrid systems combining AOPs with biological or adsorption processes gain prominence, as evidenced by studies reporting contaminant removal efficiencies above 90% through the integration of photocatalysis with persulfates. The emergence of antibiotics as a relevant term coincides with research achieving degradation efficiencies of 99% for ofloxacin within 20 min using photo-Fenton systems. Journals such as *Chemosphere*, with 1268 total citations and an average of 317 citations per article [Table 4], have been key vehicles for disseminating these applied advances, particularly through reviews such as the one on synthetic dye removal, which has accumulated 1046 citations [52]. The projection for 2025–2026 suggests convergence toward advanced oxidation processes, persistent organic pollutants, and environmental remediation as thematic cores, indicating that the field has reached a phase of integration where attention is directed toward applying mature technologies to specific problems. This trend aligns with the profile of consolidated authors such as Oturan, with 4336 total citations and 188.52 citations per year [Table 2], and Nidheesh, with 1678 citations and 209.75 citations per year [Table 2], who continue to be key references as the field specializes in specific contaminants. The persistent presence of photocatalysis across all three periods confirms its role as a backbone technology, supported by journals such as *Applied Catalysis B: Environmental*, which, with 1077 total citations and an average of 359 citations per article [Table 4], has been a fundamental outlet for these developments, including the solar photocatalysis review with 744 citations [30].

The evolution from fundamental concepts to specific applications reflects the maturity of the field, where basic mechanisms (hydroxyl radicals and mineralization) have been given a way to more complex concerns such as antibiotic removal and treatment of real wastewater. This trajectory is consistent with the geographic distribution observed in Table 3, where countries such as Spain, with 20,682 total citations and an average of 1378.8 citations per article, and France, with 6381 total citations and an average of 797.62, have led fundamental research, while China’s expansion, with 11,290 total citations and an average of 594.21, drives toward practical applications [Table 3]. The emergence of terms such as adsorption in the recent period is also reflected in works such as Gaur’s study on bioremediation, which has accumulated 398 citations [32], indicating the integration of multiple technological approaches. Figure 5 visualizes the trajectory of the field from mechanistic foundations toward a phase of application and specialization, where advanced oxidation processes—particularly photocatalysis and Fenton systems—are consolidated as mature tools for environmental remediation. This temporal evolution, consistent with citation patterns observed in previous tables, confirms that AOP research for POPs is moving toward integrated solutions where sustainability, efficiency under real conditions, and the elimination of specific contaminants define the boundaries of knowledge.

From an industrial perspective, two practical questions arise from the data. First, which AOP families are most used and effective? The analysis confirms that solar photocatalysis and electrochemical processes (including electro-Fenton and anodic oxidation) are the most widely reported and consistently achieve degradation efficiencies above 85–90% for persistent organic pollutants, as shown in the most cited works (Table 1) and the author impact analysis (Table 2). Second, what is the greatest practical potential for industrial application? The literature points to cascade systems (AOP followed by biological treatment) as the most economically viable strategy, since partial mineralization of POPs into biodegradable intermediates reduces overall treatment costs. Regarding operational factors, coupling AOPs with renewable energy (e.g., solar-powered photocatalysis or wind-powered electrochemical reactors) can lower operational energy costs by approximately 60% based on recent pilot studies [24]. Furthermore, plasma-based AOPs offer a modular design and additive-free operation, making them suitable for decentralized industrial wastewater treatment without chemical handling risks [53,54]. These insights provide a direct roadmap for industrial decision-makers seeking to implement AOP technologies.

## 3. Future Research Trends in AOPs for POP Degradation

The comprehensive bibliometric analysis conducted in this study allows for the identification of not only the current state but also a clear projection of the strategic and emerging research lines that will define the future development of AOPs applied to POPs. These trends are fundamentally aimed at overcoming the main barriers to industrial implementation: energy efficiency, comprehensive sustainability, and robust scalability.

### 3.1. AOPs Coupled with Renewable Energy and Energy Optimization

The transition towards decarbonizing remediation processes stands as an unavoidable priority. Future research will intensely focus on coupling AOPs with renewable energy sources, such as next-generation solar photocatalysis and electrochemical systems powered by wind or photovoltaic energy. This will not only reduce the carbon footprint but also lower operational costs. Pioneering studies, such as that by N Lotha et al. (2024) [24], have already highlighted the viability of these hybrid systems, demonstrating that combining solar photocatalysis with membrane reactors can achieve 95% removal of textile dyes while reducing specific energy consumption by 60% compared to conventional grid-powered AOPs. Furthermore, the optimization of modular reactors to maximize light capture or mass transfer will be an intensive area of development, aiming to achieve quantum efficiencies exceeding 40% under real operating conditions.

### 3.2. Development of Intelligent and Multifunctional Materials and Catalysts

Innovation in advanced materials will continue to be the driving force behind improvements in AOP efficiency. The trend is moving away from homogeneous catalysts towards stable, selective heterogeneous systems. An emergence in research on atomically dispersed carbocatalysts (such as metal–metal dual-site catalysts), metal–biochar hybrid nanocomposites, and visible-light-sensitive photocatalysts with tunable broad bandgaps is anticipated. These materials aim not only for high activity but also for properties such as self-cleaning, in situ regeneration, and fouling resistance. For example, emerging work on recyclable magnetic catalysts would allow for their easy separation from the treated effluent, reducing downstream costs and the risk of secondary contamination by nanoparticles. Research like that of Qiu et al. (2024) [13] on dimensionally stable titanium-based anodes already points to this future, reporting that nanostructured electrodes with mixed-oxide coatings maintain catalytic activity above 90% for over 500 h of continuous operation in degrading chlorinated herbicides.

### 3.3. Integration of Hybrid Processes and Cascade Treatment Approaches

The future of POP remediation does not lie in a single technology but in the intelligent synergy between different processes. Research will be directed toward designing cascade treatment systems that combine an AOP (such as electro-Fenton or persulfate activation) as a pre-treatment, followed by a biological or membrane filtration stage. This approach seeks to mineralize POPs into more biodegradable intermediates, which can then be completely removed by specialized microbial consortia at a lower cost. Studies such as that by Soto-Verjel et al. (2022) [6] already demonstrate that combining catalytic oxidation with membrane bioreactors can improve effluent biodegradability by over 50% and achieve combined removals of 99% of organochlorine pesticides, offering a holistic and economically viable solution for complex agricultural effluents.

### 3.4. Life Cycle Assessment, Byproduct Toxicity, and Sustainability

A critical trend will be the shift in focus from mere degradation efficiency towards a comprehensive sustainability assessment. This implies conducting a full Life Cycle Assessment (LCA) of AOP technologies, considering catalyst synthesis, energy consumption, and waste management. Concurrently, there will be increasing emphasis on identifying and assessing the toxicity of degradation byproducts, using advanced chemical analysis tools and bioassays. Future research must ensure that the “solution” does not create greater environmental problems by evaluating the acute and chronic ecotoxicity of treated effluents. “Green chemistry” principles applied to AOPs will drive the design of processes that minimize the use of hazardous reagents and maximize atom economy. In this sense, Liu et al. (2023) [16], in their review on persulfate systems, highlighted the critical need for studies that map the degradation pathways and toxicity of intermediates formed during the oxidation of complex POPs like PCBs to avoid generating byproducts that are even more toxic than the original contaminant.

### 3.5. Digitalization, Modeling, and Intelligent Process Control

Finally, the Fourth Industrial Revolution will achieve POP remediation through the integration of digitalization tools. The extensive use of artificial intelligence and machine learning is foreseen to optimize operational parameters in real-time, predict the degradation kinetics of new contaminants, and design catalysts in silico. Computational Fluid Dynamics (CFD) modeling will be crucial for designing scalable, high-efficiency reactors. This precision engineering approach will enable a faster, more reliable, and cost-effective transition from optimal laboratory conditions to pilot plants and the industrial scale. Works such as that by Li et al. (2023) [25] on VOC removal, albeit in a different matrix, illustrate the potential of these predictive models to reduce the development and optimization times for new AOP systems, ultimately closing the gap between scientific innovation and the widespread technological application required to mitigate the global POP crisis.

### 3.6. Plasma-Based AOPs: An Emerging Frontier

Non-thermal plasma-based advanced oxidation processes represent an emerging frontier in the degradation of persistent organic pollutants. Unlike conventional AOPs that require external reagents, plasma generates in situ a complex mixture of reactive species—including hydroxyl radicals (OH), ozone, hydrogen peroxide, and UV radiation—through electrical discharges at atmospheric pressure and ambient temperature, enabling simultaneous attack on POPs through multiple pathways. Significant contributions to this field come from Petrović and collaborators. In 2021, they demonstrated that electrochemically synthesized molybdenum oxides (MoO_2_ and MoO_3_) increase the degradation efficiency of Reactive Blue 19 dye by 45–50% using pulsed corona plasma, reducing energy consumption while maintaining catalytic activity after five reuse cycles [53]. Subsequently, they developed a plasma-modified CeO_2_ catalyst that significantly enhanced both plasma and photocatalytic degradation of the same pollutant, demonstrating that plasma can serve both as a degradation tool and as a catalyst modification technique [54].

These findings align with recent studies reporting complete sulfamethoxazole removal within 25 min using dielectric barrier discharge plasma, with total mineralization being achieved in 6 h and non-toxic transformation products. Key advantages include: operation without chemical additives, compatibility with renewable energy sources, capability to treat emerging contaminants such as PFAS, and modular designs suitable for decentralized wastewater treatment. Future research should focus on optimizing reactor geometry, developing stable catalysts for plasma environments, and validating performance with real industrial effluents.

## 4. Methodology

**Figure 6:**
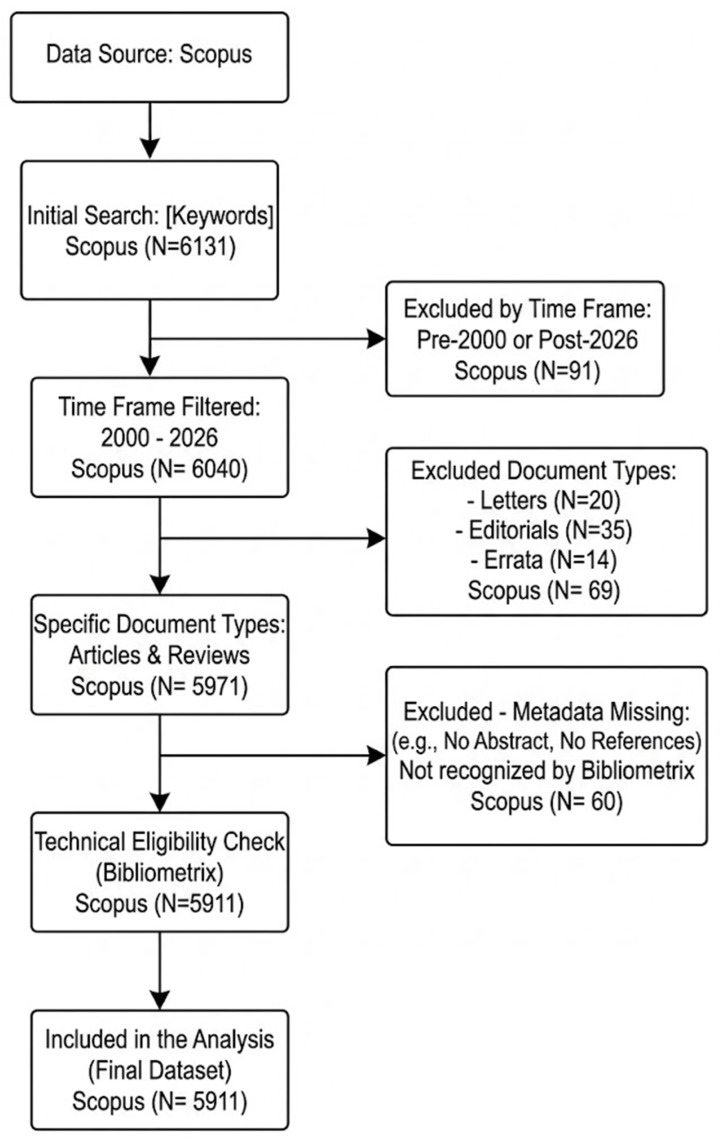
Flowchart of the document selection process for the Scopus-based analysis (2000–2025). Excluded document types: editorials, notes, errata, letters to the editor, conference reviews, short surveys, and duplicate records.

This research was based on a systematic analysis of the scientific literature on the degradation of POPs through AOPs. Data collection was carried out using the Scopus database, selected for its breadth, multidisciplinary coverage, and the quality of its metadata. The search strategy employed a combination of key terms related to POPs and AOPs: TITLE-ABS-KEY (((“persistent organic pollutant*” OR “POP*” OR “PCB*” OR “PAH*” OR “PFAS” OR “dioxin*” OR “organochlorine pesticide*”) AND (“advanced oxidation” OR “AOP*” OR “fenton” OR “photocataly*” OR “ozon*” OR “persulfate” OR “sonolysis” OR “electrochemical oxidation”) AND (“degrad*” OR “remov*” OR “mineraliz*”))) AND PUBYEAR > 1999 AND PUBYEAR < 2027. From the initial set of retrieved documents, a filtering process was applied to ensure the analysis exclusively focused on substantive scientific contributions. The following document types were systematically excluded: editorials, notes, errata, letters to the editor, conference reviews, short surveys, and duplicate records identified by DOIs or title matching. Only research articles, full reviews, and book chapters were retained for final analysis. This exclusion criteria aligns with standard bibliometric practices to avoid distortion of impact indicators by non-citable or ephemeral documents [17,19]. Data processing and analysis were carried out using the R Studio programming environment, specifically with the bibliometrix package, enabling robust statistical analyses. Productivity indicators (number of publications per year, country, author, and institution) and impact indicators (total citations, citations per year, and h-index) were quantified. To visualize and explore the intellectual structure and collaborative dynamics of the field, specialized tools were employed. VOSviewer was used for mapping and constructing networks of keyword co-occurrence, co-authorship, and co-citation, thereby identifying the main thematic clusters and patterns of international collaboration. Complementarily, the Plotly library in R was used to generate interactive graphs and dashboards, facilitating clear and dynamic communication of quantitative findings and temporal trends. This comprehensive methodology not only enabled diagnosis of the state of the art but also allowed for visualization of the thematic and geographical connections shaping this rapidly evolving field of research (Figure 6).

## 5. Conclusions

Pollution by persistent organic pollutants represents an environmental and public health crisis on a global scale due to their toxicity, persistence, and capacity for bioaccumulation in the food chain. Given the limitations of conventional methods, which are often costly or generate hazardous byproducts, AOPs have emerged as effective technological solutions for the terminal destruction of these compounds. Unlike previous bibliometric analyses that focus on general AOP applications in wastewater or on single technologies such as photocatalysis or electro-Fenton, the present study provides a differentiated contribution explicitly built upon four novelty pillars: first, the specific targeting of persistent organic pollutants (POPs) as a priority contaminant class rather than generic organic pollutants; second, the most up-to-date temporal coverage (2000–2026), including literature from the last two years not captured in earlier bibliometric reports; third, the integration of emerging research frontiers—namely, plasma-based AOPs and digitalization tools (artificial intelligence, computational fluid dynamics, and machine learning)—for process optimization and reactor design; and fourth, the proposal of a strategic roadmap that bridges laboratory innovations with industrial scalability, addressing the critical gap between fundamental research and real-world application. These four elements collectively distinguish our work from previous reviews and bibliometric reports, offering a more comprehensive and forward-looking perspective for researchers, policymakers, and industry stakeholders. The methodology consisted of a quantitative analysis using the Scopus database, from which 5911 documents were retrieved and processed with specialized tools such as R Studio (bibliometrix) and VOSviewer. The results reveal exponential growth in research within the field, with an annual growth rate exceeding 18%. This expansion reflects accelerated scientific interest, driven both by environmental urgency and the tightening of international regulations on POPs.

In terms of geographic productivity and scientific impact, China leads in publication volume with 109 documents, followed by India and the United States. However, the highest impact per article is found in Spain and France, with averages of 217.5 and 213.5 citations respectively, underscoring the influence of their researchers as theoretical and methodological benchmarks. Authors such as Malato (Spain) and Oturan (France) act as central nodes of international collaboration, accumulating thousands of citations in areas such as solar photocatalysis and electro-Fenton processes. The analysis of applied technologies confirms that solar photocatalysis and electrochemical processes are the most promising families of AOPs, consistently reporting degradation efficiencies above 85–90%. Thematically, wastewater treatment is identified as the “motor theme” driving the field, while advanced catalyst design has evolved into a niche technical specialization. Journals such as *Chemosphere* and *Science of the Total Environment* have consolidated as the primary dissemination channels for this research. Finally, the study proposes a strategic roadmap to bridge the gap between laboratory innovation and industrial applications. Future directions should prioritize coupling AOPs with renewable energies to decarbonize remediation processes, the development of smart and magnetically recoverable materials, and the integration of hybrid cascade systems. In addition, the importance of digitalization is highlighted through the use of artificial intelligence and computational fluid dynamics models to optimize large-scale reactor design.

Future work should focus on: (1) coupling AOPs with renewable energy to decarbonize remediation; (2) developing intelligent and multifunctional catalysts (e.g., dual atomic sites and magnetic materials) that combine high activity, stability, and ease of recovery; (3) integrating hybrid cascade processes (AOP + biological) to address complex effluents in a holistic and economical manner; (4) conducting comprehensive sustainability assessments through life cycle analysis (LCA) and an evaluation of byproduct ecotoxicity, ensuring that solutions do not create new environmental liabilities; and (5) advancing digitalization in the field, employing artificial intelligence and computational modeling to optimize reactor design and predict kinetics, thereby accelerating the scale-up from laboratory research to massive, sustainable industrial applications.

## Figures and Tables

**Table 1:** Most cited publications in the field of degradation of persistent organic pollutants through advanced oxidation processes (2000–2026).

#	Title	Citations	Citations/Year	Year	Journal	First Author	Document Type
**1**	Decontamination and disinfection of water by solar photocatalysis: Recent overview and trends [26].	2664	222	2009	*Catalysis Today*	Malato	Review
**2**	Advanced oxidation processes in water/wastewater treatment: Principles and applications. A review [27].	1995	285	2014	*Critical Reviews in Environmental Science and Technology*	Oturan	Review
**3**	An overview on the removal of synthetic dyes from water by electrochemical advanced oxidation processes [28].	1046	348.67	2018	*Chemosphere*	Nidheesh	Review
**4**	An overview of photocatalytic degradation: photocatalysts, mechanisms, and development of photocatalytic membrane [29].	969	161.5	2020	*Environmental Science and Pollution Research*	Koe	Review
**5**	Photocatalysis with solar energy at a pilot-plant scale: An overview [30].	744	39.16	2002	*Applied Catalysis B: Environmental*	Malato	Review
**6**	Electrochemical oxidation remediation of real wastewater effluents—A review [31].	681	227.35	2018	*Process Safety and Environmental Protection*	Garcia-Segura	Review
**7**	Recent advances in the bioremediation of persistent organic pollutants and its effect on environment [32]	398	132.67	2018	*Journal of Cleaner Production*	Gaur	Review
**8**	Laccases and peroxidases: The smart, greener and futuristic biocatalytic tools to mitigate recalcitrant emerging pollutants [33].	296	49.33	2020	*Science of the Total Environment*	Morsi	Review
**9**	Photocatalytic decontamination and disinfection of water with solar collectors [34].	262	18.71	2007	*Catalysis Today*	Malato	Article
**10**	A Review Study on Sulfate-Radical-Based Advanced Oxidation Processes for Domestic/Industrial Wastewater Treatment: Degradation, Efficiency, and Mechanism [35].	256	42.67	2020	*Science of the Total Environment*	Rizzo	Review

**Table 2:** Most relevant authors in the field of POP degradation using AOPs, ordered by scientific impact (2000–2026).

Author	Publications	Citations	Citations per Year	H-Index	G-Index	Initial Year	Country	Institution
Zhang, H.	13	456	76.00	8	13	2020	China	Chinese Academy of Sciences
Zhang, X.	10	130	26.00	4	10	2021	China	Harbin Normal University
Zhang, Y.	10	330	27.50	7	10	2013	China	Zhejiang University
Oturan, M.A.	8	4336	188.52	8	8	2003	France	Université Paris-Est Marne-la-Vallée
Li, X.	8	343	42.88	6	8	2018	China	Ningxia University
Yang, Y.	7	271	45.17	5	7	2020	China	Harbin Institute of Technology
Liu, Y.	7	116	29.00	5	7	2022	China	Tsinghua University
Nidheesh, P.V.	7	1678	209.75	6	7	2018	India	CSIR-National Environmental Engineering Research Institute (NEERI)
Garcia-Segura, S.	7	1020	127.50	7	7	2018	USA	Arizona State University
Malato, S.	6	4741	197.54	6	6	2002	Spain	CIEMAT

**Table 3:** Scientific output by country in POP degradation by AOPs (2000–2026).

Country	Publications	Total Citations	H-Index	Average Citations	Top Institution	Institution Publications	Avg Citations per Institution	H-Index
China	19	11,290	57	594.21	MOE Key Laboratory of Pollution Processes and Environmental Criteria, Nankai University	3	501.67	10
Spain	15	20,682	35	1378.8	CIEMAT-Plataforma Solar de Almería	5	3693.2	20
India	13	4079	25	313.77	Department of Civil Engineering, Indian Institute of Technology Kharagpur	3	126.33	3
Brazil	8	988	18	123.5	Department of Materials Engineering, Federal University of São Carlos (UFSCar)	1	96	4
France	8	6381	19	797.62	Laboratoire Géomatériaux et Environnement (LGE), Université Paris-Est	5	1173	11
United States	8	1269	8	158.62	Nanosystems Engineering Research Center (NASCENT), University of Texas at Austin	3	247	3
Australia	5	3289	7	657.8	University of Queensland	3	1057	3
Taiwan	4	116	7	29	Department of Environmental Engineering, National Cheng Kung University (NCKU)	3	23.33	3
Malaysia	3	5754	10	1918	Department of Chemical Engineering, Universiti Teknologi PETRONAS	2	2763	6
Turkey	3	124	8	41.33	Department of Chemistry, Ankara University	2	50	6

**Table 4:** Bibliometric performance of leading journals in research on AOPs applied to POPs (2000–2026).

	Journal	Publications	Total Citations	H- Index	Average Citations	Most Cited Paper	Citations	Type
1	*Science of the Total Environment*	5	700	5	140	Laccases and peroxidases: the smart, greener and futuristic biocatalytic tools to mitigate recalcitrant emerging pollutants [33].	296	Review
2	*Journal of Hazardous Materials*	5	470	5	94	Selective electrochemical H_2_O_2_ generation and activation on a bifunctional catalyst for heterogeneous electro-Fenton catalysis [48].	225	Article
3	*Environmental Science and Pollution Research*	5	1234	4	246.8	An overview of photocatalytic degradation: photocatalysts, mechanisms, and development of photocatalytic membrane [29].	969	Review
4	*Chemical Engineering Journal*	4	557	4	139.25	Bio-electro-Fenton processes for wastewater treatment: Advances and prospects [49].	192	Review
5	*Chemosphere*	4	1268	4	317	An overview on the removal of synthetic dyes from water by electrochemical advanced oxidation processes [28]	1046	Article
6	*Water, Air, and Soil Pollution*	3	45	3	15	Photocatalytic Treatment of Olive Oil Mill Wastewater Using TiO_2_ and Fe_2_O_3_ Nanomaterials [48].	20	Article
7	*Applied Catalysis B: Environmental*	3	1077	3	359	Photocatalysis with solar energy at a pilot-plant scale: An overview [30].	744	Review
8	*Journal of Environmental Management*	2	242	2	121	A review on treatment of petroleum refinery and petrochemical plant wastewater: a special emphasis on constructed wetlands [50].	183	Review
9	*Journal of Water Process Engineering*	2	218	2	109	Evaluation of advanced oxidation processes (AOP) using O_3_, UV, and TiO_2_ for the degradation of phenol in water [51].	158	Article
10	*Process Safety and Environmental Protection*	2	729	2	364.5	Electrochemical oxidation remediation of real wastewater effluents—A review [31]	681	Review

## Data Availability

The original contributions presented in this study are included in the article. Further inquiries can be directed to the corresponding author.
